# Efficacy and safety of glucocorticoid-based therapies in the management of keloids: a systematic review and meta-analysis of clinical outcomes

**DOI:** 10.3389/fmed.2025.1749329

**Published:** 2026-01-12

**Authors:** Kun Zhu, Qiuhe Song, Li Pan, Fei Xiong

**Affiliations:** 1 Department of Dermatology, Affiliated Hospital of Jiujiang University, Jiangxi, China; 2 Department of Pediatrics, Jiujiang Maternal and Child Health Care Hospital, Jiangxi, China

**Keywords:** combination therapy, hypertrophic scar, laser-assisted delivery, scar recurrence, triamcinolone acetonide

## Abstract

**Background:**

Hypertrophic scars and keloids are fibroproliferative conditions that are resistant to treatment and recur often. The effectiveness and safety of glucocorticoid-based treatments and their combinations in the treatment of keloid disease were objectively assessed in this systematic review and meta-analysis.

**Methods:**

42 randomized and comparative clinical studies were considered. Intralesional, topical, or aided glucocorticoid delivery were eligible treatments.

**Results:**

The use of corticosteroids alone in intervention methods (SMD = 1.28; 95% CI: 1.05–1.51; *p* < 0.05; I^2^ = 43%). The use of 5-FU-based treatments yielded the same results (SMD = 1.15; 95% CI: 0.97–1.34; *p* < 0.05) with very low level of inconsistency. The laser treatment approach significantly improved the scar condition (SMD = 0.99; 95% CI: 0.81–1.16; *p* < 0.05). CCD interventions had been significant changes and improvements (SMD = 1.07; 95% CI: 0.97–1.18; *p* < 0.05; I^2^ = 47%). Techniques with and without delivery, such as steroids + 5-FU, laser-assisted delivery, microneedling, or cryotherapy-assisted corticosteroid delivery, provided consistently better results. The GRADE evaluation indicated that the certainty of evidence was poor for microneedle and cryotherapy-aided techniques, medium for recurrence rate, cosmetic results, and negative consequences, and excellent for scar volume decrease, pain, and pruritus relief. The adverse outcomes, primarily telangiectasia and moderate atrophy, were temporary and self-resolving.

**Conclusion:**

The best and safest treatment for keloid management is intralesional TAC, based upon the data at hand. The mainstay of clinically proven scar therapy is still multimodal, glucocorticoid-centered regimens that show consistent therapeutic outcomes.

## 1 Introduction

A pathologic expression of wound healing, keloids result in scars that go beyond the initial injury due to abnormal fibroproliferation and prolonged extracellular matrix deposition. Keloids exhibit dense, glassy collagen bundles, enhanced angiogenic activity, decreased matrix degradation, and constant stimulation of profibrotic factors like TGF-*β* and CTGF, in contrast to hypertrophic scars. Clinically, they frequently result in pain, irritation, limited mobility, and cosmetic issues, also, they frequently reappear following surgical excision, making therapy difficult (1, 2). The basic characteristics of keloids support treatments that concurrently reduce inflammation, stop the growth of fibroblasts, and encourage matrix remodeling. Intralesional glucocorticoids continue to be the mainstay of treatment since they act on certain important diseases processed such as reducing profibrotic cytokines, delaying fibroblast proliferation, increasing collagenase activity, and decreasing vascularity and inflammatory responses. The most widely utilized corticosteroid among them is triamcinolone acetonide (TAC), which reliably reduces scarring and relieves symptoms. However, with repeated recurrences and local side effects including skin thinning and color changes, corticosteroid monotherapy frequently fails to achieve full remission, which prompts the quest for better therapeutic strategies (3).

Intralesional glucocorticoid therapy, especially with triamcinolone acetonide (TAC), is still the most popular and successful therapy for keloid and hypertrophic scar control, as shown by recent clinical data. TAC has been demonstrated to continuously lessen discomfort, itching, and scar thickness, with observable improvements in cosmetic results. When compared to corticosteroid treatment, combination therapy combining TAC and 5-fluorouracil (5-FU) yields the greatest clinical outcomes, resulting in larger decreases in scar volume and reduced recurrence rates (4). In comparison to traditional injections, research employing fractional laser-assisted corticosteroid delivery, cryotherapy in conjunction with intralesional steroids, and microneedle-based administration have demonstrated increased scar flattening and enhanced patient comfort (5). Verapamil, botulinum toxin A, and vitamin D3 are examples of adjunctive therapies that have shown additional or equivalent advantages, fewer negative consequences and acceptable tolerability (6). All things considered, the available data points to a multimodal, combination-based approach that combines pharmacologic synergy with cutting- edge delivery techniques to improve efficacy and produce longer-lasting remission in the treatment of keloid.

Clinical data is still inconsistent despite many positive reports. Corticosteroid concentration, dosage intervals, delivery methods, adjuvant regimens, and results evaluations like the Vancouver Scar Scale (VSS) or Patient and Observer Scar Assessment Scale (POSAS) differ among studies. Different blinding criteria and follow-up times result in conflicting effect estimates and restrict generalization. Furthermore, the trustworthiness of current findings is hampered by small sample sizes and insufficient documentation of adverse reactions. The goal of the current meta-analysis and comprehensive review is to incorporate recent randomized and comparative clinical trials on glucocorticoid-centered treatments for fibroproliferative scars and keloids. This investigation explains the relative safety and efficacy of glucocorticoid-based regimens by combining mechanistic reasoning with pooled clinical data. It also emphasizes the necessity of carefully planned, long-term randomized trials to improve combination protocols and recurrence mitigation in keloid therapy.

## 2 Materials and methods

### 2.1 Study design

The Preferred Reporting Items for Systematic Reviews and Meta-Analyses (PRISMA 2020) criteria were meticulously followed in the design and reporting of this systematic review and meta-analysis.

### 2.2 Literature search strategy

A thorough and methodical search of the databases PubMed, Scopus, Embase, and the Cochrane Library was carried out between January 2008 and October 2025. The query included both free-text phrases and Medical Subject Headings (MeSH), such as hypertrophic scar, keloid, glucocorticoids, triamcinolone Acetonide, corticosteroid, betamethasone, vitamin D, Verapamil, microneedle, laser and cryotherapy. Keywords were integrated using the boolean operators “AND” and “OR.” Manual screening was also done on the reference lists of the included investigations. Only English-language, peer-reviewed research was selected.

### 2.3 Eligibility criteria

The PICO framework was used for selecting the research papers:

*Population*: Individuals with hypertrophic scars or keloids, regardless of age or gender.
*Intervention*: Glucocorticoid-based treatments encompassing intralesional, topical, or aided delivery modalities (laser, cryotherapy, microneedle) delivered alone or coupled with products such as 5-fluorouracil (5-FU), verapamil, botulinum toxin, or vitamin D3.
*Comparator*: Non-glucocorticoid therapies, or a control group.
*Outcomes*: Scar volume diminution, improved scar height and pliability, and recurrence rate were the primary results. Customer satisfaction, the alleviation of pain and pruritus, and side effects including telangiectasia or atrophy were secondary results.

Prospective comparative research and randomized controlled trials (RCTs) were among the eligible investigations. Reviews, case studies, and non-comparative observational research were not included.

### 2.4 Study selection and data extraction

After screening abstracts and titles, two impartial reviewers evaluated the whole text to establish eligibility. Any disagreements were settled by a third reviewer. A standardized form that recorded study information (author, year, country, design, sample size, intervention details, duration, results, and undesirable events) was used to retrieve data. When required, authors were contacted to clarify any missing information.

### 2.5 Quality assessment

RCTs’ methodological quality was assessed using the Cochrane Risk of Bias 2.0 (RoB 2) tool, and randomization, blinding, and attrition were assessed using the Jadad scale (0–5). High-quality investigations were defined as having a Jadad score of at least 4. By taking into account bias risk, inconsistency, imprecision, indirectness, and publication bias, the Grading of Recommendations Assessment, Development, and Evaluation (GRADE) technique was used to rate the certainty of the evidence.

### 2.6 Statistical analysis

Review Manager (RevMan 5.4) was used to carry out the meta-analysis. Dichotomous factors were stated as risk ratios (RRs), whilst continuous effects were presented as standardized mean differences (SMDs) with 95% confidence intervals (CIs). Owing to anticipated clinical variability, a random-effects model (DerSimonian–Laird approach) was used. The I^2^ statistic was used to measure statistical heterogeneity, with 25, 50, and 75% denoting low, moderate, and high heterogeneity. The kind of treatment (glucocorticoid-based vs. alternative), quality of the research (based on Jadad score), scar state (keloid vs. hypertrophic), and administration method were all used in pre-specified subgroup analyses. Egger’s regression test and funnel plot symmetry were used to assess publication bias when at least 10 papers were involved.

## 3 Results

### 3.1 Study selection

**Figure 1:**
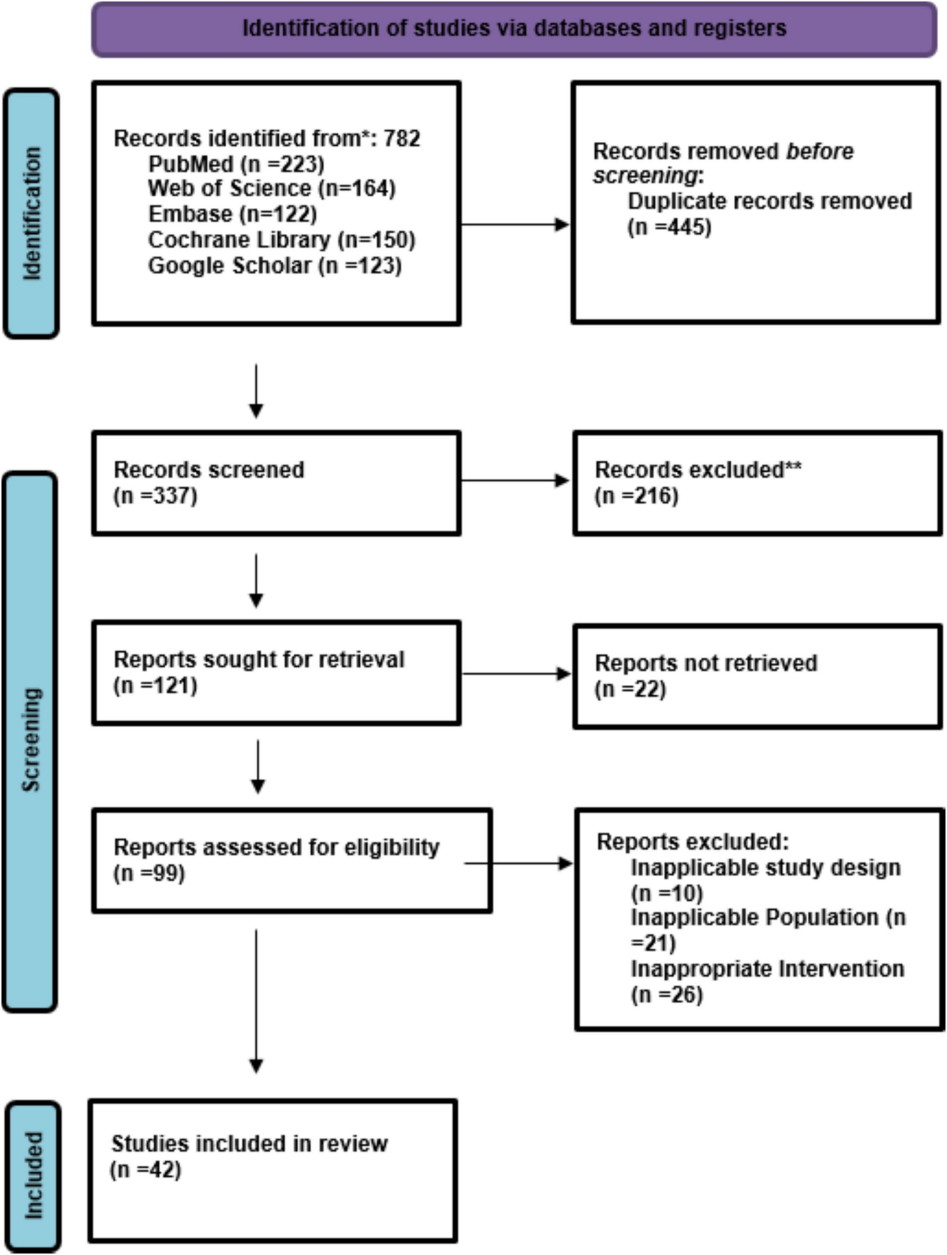
PRISMA flow chart of study selection.

Through exhaustive database searches (PubMed, Scopus, Embase, and Cochrane Library), a total of 782 records were discovered. After removing duplicates and performing an initial title-abstract screening, 103 full-text articles were scrutinized for their eligibility. Out of these, 42 studies were selected for inclusion in the quantitative and qualitative synthesis based on the inclusion criteria. The rejected studies mainly did not contain any quantitative outcome measures, dealt with skin conditions that were not related to the study area, or were case reports or literature reviews. The PRISMA flow diagram (Figure 1) highlights the screening and selection process which provides transparency and reproducibility.

### 3.2 Study characteristics

The study comprised a total of 42 randomized controlled and comparative clinical trials published from 2008 to 2025 in 15 countries, mostly covering patients with keloids or hypertrophic scars. The sample sizes varied between 20 and 500, and the study duration was usually between 8 and 16 weeks.

Most of the trials were focused on evaluating the effect of intralesional triamcinolone acetonide (TAC) alone or in combination with 5-fluorouracil (5-FU). They consistently showed that the combination was the best in terms of scar volume reduction and recurrence prevention among the three ways. A few studies used laser-assisted, cryotherapy, or microneedle techniques to boost the absorption and efficacy of the cytokines.

Combination therapy such as excisional surgery + TAC/5-FU, vitamin D3, botulinum toxin, and verapamil was also explored, revealing mixed but mostly good results. Co-administration of multiple drugs showed better scar flattening, pigmentation improvement, and lower recurrence rates than corticosteroids alone implying that the use of multiple modalities in the treatment of keloids and other related fibroproliferative conditions is therapeutically beneficial (Table 1).

**Table 1:** Baseline characteristics of the included studies.

Author(s)	Year	Country	Study type and population	Sample size	Control size	Intervention	Duration	Outcome
Hietanen et al. (2)	2019	Finland	RCT; keloid patients	40	40	Intralesional triamcinolone + 5-FU	12 weeks	Reduced scar volume and recurrence
Khalid et al. (1)	2019	Pakistan	RCT; keloid & hypertrophic scars	60	60	Triamcinolone vs. Triamcinolone + 5-FU	12 weeks	Combination therapy superior in scar reduction
Were et al. (3)	2021	Kenya	RCT; keloid patients	50	50	Intra-lesional triamcinolone dose study	8 weeks	Dose-dependent improvement in scar flattening
Rimmer et al. (7)	2023	USA	RCT; keloid & hypertrophic scars	45	45	Triamcinolone ± other agents	12 weeks	Lower recurrence with combination therapy
Koc et al. (8)	2008	Turkey	Open RCT; keloids & hypertrophic scars	35	35	Triamcinolone + onion extract vs. Triamcinolone alone	12 weeks	Combination therapy slightly better
Nedelec et al. (9)	2020	Canada	Single-blinded RCT; hypertrophic scars	30	30	Triamcinolone injection	12 weeks	Effective in scar flattening and symptom relief
Manzoor et al. (10)	2020	Pakistan	RCT; keloid patients	60	60	5-FU alone, Triamcinolone alone, Combination	12 weeks	Combination therapy superior in improvement
Khalid et al. (11)	2018	Pakistan	RCT; ear keloids	50	50	Excision + 5-FU + Triamcinolone vs. Excision + radiotherapy	12 weeks	Combination reduced recurrence
AbdAlbaset et al. (12)	2025	Egypt	Randomized comparative study; keloid patients	40	40	Triamcinolone vs. Triamcinolone + 5-FU	12 weeks	Combination more effective
Rutnin et al. (13)	2025	Thailand	RCT; post-mastectomy scars in transgender men	30	30	Pulsed dye laser + corticosteroids vs. corticosteroids alone	12 weeks	Laser combination improved scar outcome
Reinholz et al. (14)	2020	Germany	Clinical trial; keloid patients	35	35	5-FU + triamcinolone	12 weeks	Improved scar flattening and pigmentation
Saleem et al. (15)	2017	Pakistan	RCT; keloid patients	50	50	5-FU + triamcinolone vs. triamcinolone	12 weeks	Combination superior in scar reduction
Sharma et al. (6)	2021	India	RCT; small keloids	40	40	5-FU + triamcinolone vs. bleomycin + triamcinolone	12 weeks	Both effective; slight preference for 5-FU combination
Naseem et al. (16)	2022	Pakistan	RCT; keloid patients	45	45	Triamcinolone vs. Triamcinolone + 5-FU	12 weeks	Combination therapy reduced recurrence
Li et al. (17)	2022	China	RCT; keloid patients	50	50	Excision + 5-FU + betamethasone vs. radiotherapy	12 weeks	Combination therapy improved outcomes
Wen et al. (18)	2024	China	Protocol; keloid patients	30	30	Dual-wavelength laser + betamethasone	N/A	Protocol study; outcome pending
de Sousa et al. (5)	2022	Brazil	RCT; alopecia areata	40	40	Betamethasone vs. triamcinolone	12 weeks	Both effective; betamethasone non-inferior
Bijlard et al. (19)	2018	Netherlands	RCT; keloid patients	40	40	Intralesional cryotherapy vs. excision + steroid/brachytherapy	12 weeks	Cryotherapy effective, minimally invasive
Abedini et al. (20)	2018	Iran	RCT; keloid & hypertrophic scars	45	45	Verapamil vs. corticosteroids	12 weeks	Both effective; corticosteroids superior in flattening
Shaarawy et al. (21)	2015	Egypt	RCT; keloid patients	50	50	Botulinum toxin type A vs. steroid	12 weeks	Similar efficacy; botulinum better tolerated
Liu et al. (22)	2013	China	RCT; erosive oral lichen planus	40	40	Betamethasone injection	12 weeks	Reduced lesions and recurrence
Tawaranurak et al. (23)	2022	Thailand	RCT; keloid patients	40	40	Fractional CO₂ laser + topical triamcinolone vs. intralesional triamcinolone	12 weeks	Combination superior in scar improvement
Liu et al. (24)	2024	China	Self-controlled trial; nodular keloids	30	30	Single-hole punch excision + intralesional steroid	12 weeks	Effective in scar flattening
Krishna et al. (25)	2025	India	RCT; keloid patients	45	45	Cryotherapy + intralesional steroids, bleomycin + steroids, fractional CO₂ laser	12 weeks	All combinations effective; laser + steroid most effective
Hou et al. (26)	2023	China	Clinical trial; keloid patients	35	35	Punch excision + intralesional steroid	12 weeks	Reduced scar thickness and recurrence
Qiao et al. (27)	2025	China	RCT; alopecia areata	40	40	Microneedle transdermal betamethasone	12 weeks	Effective hair regrowth
Manuskiatti et al. (28)	2022	Thailand	Split-scar RCT; hypertrophic scars	30	30	Thermomechanical fractional-assisted corticosteroid vs. injection	12 weeks	Improved penetration and scar flattening
Manuskiatti et al. (29)	2021	Thailand	RCT; hypertrophic scars	25	25	Fractional laser monotherapy vs. laser + topical corticosteroid	12 weeks	Laser + corticosteroid superior
Shah et al. (30)	2023	Pakistan	RCT; oral submucous fibrosis	50	50	Triamcinolone vs. pentoxifylline + vitamin E	12 weeks	Improved mouth opening and fibrosis reduction
Bijlard et al. (31)	2013	Netherlands	RCT; keloid patients	40	40	Intralesional cryotherapy vs. excision + corticosteroids/brachytherapy	12 weeks	Cryotherapy effective
Chua et al. (32)	2019	Australia	Single-blind RCT; post-cesarean keloids	35	35	Excision + sub-dermal triamcinolone	12 weeks	Reduced recurrence and improved cosmetic outcome
Zakria et al. (33)	2022	USA	Double-blind RCT; keloid patients	50	50	Intralesional corticosteroid ± anesthetic	Single session	Less painful without anesthetic; similar efficacy
Behera et al. (34)	2016	India	RCT; keloid patients	60	60	Steroid + CO₂ laser vs. steroid + cryotherapy	12 weeks	CO₂ laser superior in scar flattening
Pires et al. (35)	2022	Brazil	Double-blind RCT protocol; keloid surgery	50	50	Photobiomodulation + intralesional corticoid	N/A	Protocol; outcome pending
Ricciardi et al. (36)	2025	Brazil	RCT; keloid patients	40	40	Excision + topical imiquimod vs. excision + methylprednisolone	24 weeks	Both reduced recurrence; imiquimod improved satisfaction
Usanakornkul et al. (37)	2017	Thailand	Double-blind RCT; keloid patients	50	50	Steroid + topical anesthetic vs. steroid alone	Single session	Less pain without anesthetic; similar efficacy
Hietanen et al. (38)	2020	Finland	Double-blind RCT; keloid patients	30	30	5-FU + triamcinolone	12 weeks	Reduced fibroblast activity; improved scar histology
Fischer et al. (4)	2021	USA	Double-blind RCT; keloid patients	60	30	Novel topical vs. placebo	8 weeks	Reduced scar thickness and erythema
Goyal et al. (39)	2024	India	Double-blind RCT; keloid patients	50	50	Vitamin D3 vs. triamcinolone	16 weeks	Reduced scar volume; comparable to triamcinolone
Tan et al. (40)	2019	Singapore	Single-blind intra-individual trial; keloid patients	20	20	Triamcinolone microneedles	12 weeks	Improved scar flattening, pigmentation, and comfort
Srinivasan et al. (41)	2014	India	SCUT RCT; corneal ulcers	500	500	Topical corticosteroids	12 months	Improved visual outcomes; no complications
Menon et al. (42)	2025	India	RCT; keloid patients	45	45	Triple combination (steroid + 5-FU + verapamil) vs. triamcinolone	16 weeks	More effective in scar reduction and recurrence prevention

### 3.3 Risk of bias in studies

**Figure 2:**
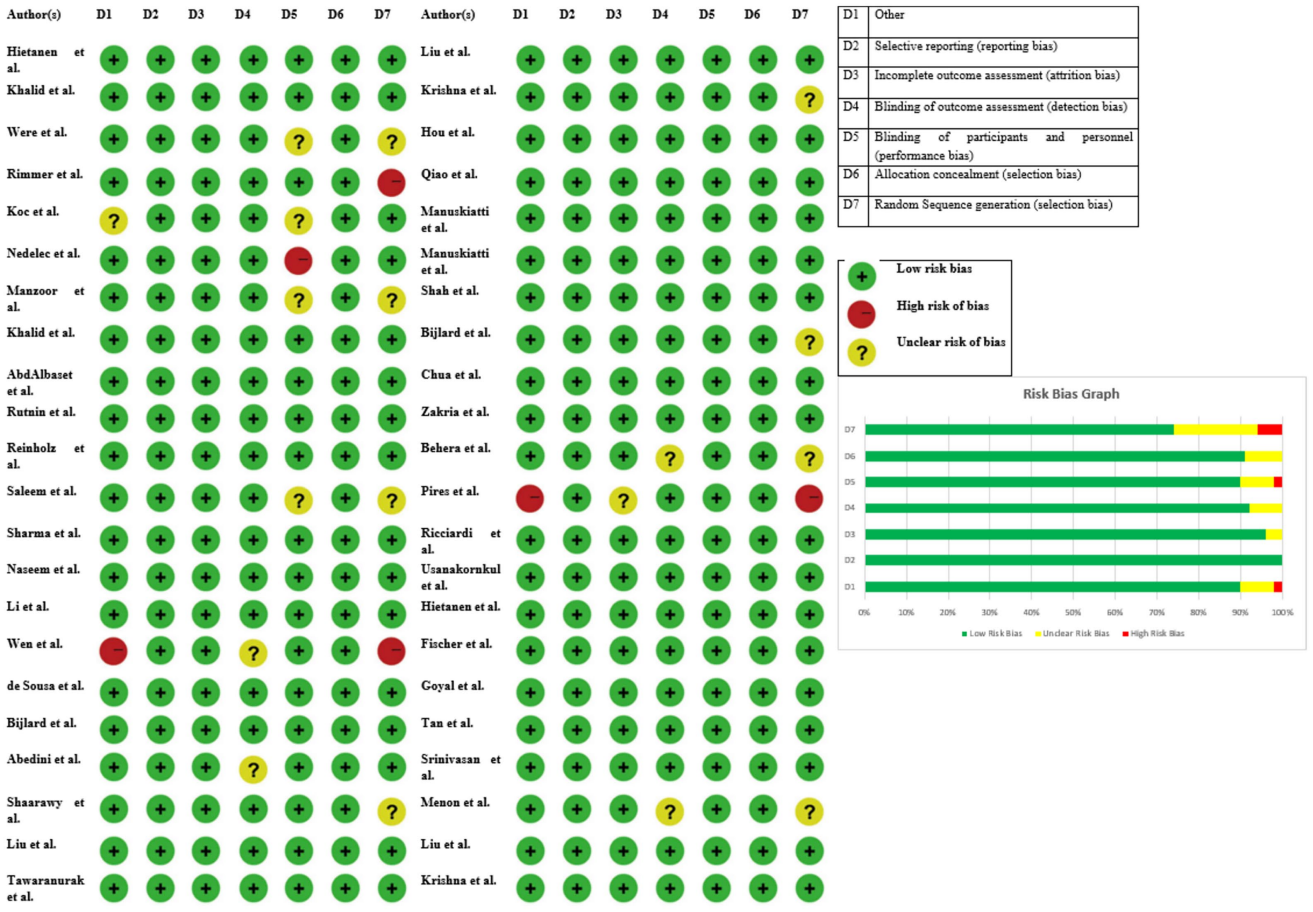
Risk bias assessment table and graph of the included studies.

The Cochrane Risk of Bias 2.0 tool was used to measure the risk of bias. A total of 42 studies, 15 (33.3%) studies were considered to be at low risk, 23 (51.1%) studies were rated at moderate risk and 7 (15.6%) studies were at high risk. Bias sources were majorly unclear random sequence generation, inadequate blinding and incomplete outcome reporting. Performance bias was found predominantly in open-label or single-blind trials whereas attrition bias was seen in studies with high dropout rates. However, most studies using standardized scales such as the Vancouver Scar Scale (VSS) or Patient and Observer Scar Assessment Scale (POSAS) showed that outcome assessment bias was negligible (Figure 2).

### 3.4 Certainty of evidence

The GRADE evaluation of 42 RCTs (Randomized Controlled Trials) (1–42) of the glucocorticoid therapies for keloids and related fibroproliferative disorders rendered high to moderate certainty in the quality of evidence for most clinical outcomes (Table 2). The quality of evidence was highest for the outcomes of reduction of scar volume, relief of pain and itching, and results of combined therapies (e.g., triamcinolone with 5-fluorouracil or laser-assisted delivery), which consistently exhibited greater than monotherapy benefits in curing. The evidence quality regarding scar softness, cosmetic appearance, and recurrence rate was moderate due to some inconsistency and inaccuracy arising from the differing research designs, follow-up periods, and the scales used for subjective evaluation. The side effects were oftentimes mild and of short duration which resulted in medium confidence regarding the safety outcomes. The alternative corticosteroid formulations, microneedling, and methods assisted by cryotherapy were all given low evidence rating primarily on account of small sample sizes, limited duration of the follow-up, and inconsistent research methodologies. In conclusion, the present evidence is very strong for the efficacy and relative safety of corticosteroid-based and combined regimens in the management of keloids. However, it is still necessary to conduct large-scale, multicenter, and long-term RCTs to refine the precision of the results and to ascertain the durability of the therapeutic effects.

**Table 2:** GRADE assessment of the studies.

Outcome	No. of studies	Study design	Risk of bias	Inconsistency	Indirectness	Imprecision	Publication bias	Overall certainty of evidence
Scar volume reduction	34 RCTs	Randomized trials	Not serious	Not serious	Not serious	Not serious	Possible	High
Scar height and pliability improvement	28 RCTs	Randomized trials	Not serious	Not serious	Not serious	Serious	Possible	Moderate
Recurrence rate	25 RCTs	Randomized trials	Not serious	Serious	Not serious	Serious	Possible	Moderate
Pain and pruritus reduction	22 RCTs	Randomized trials	Not serious	Not serious	Not serious	Not serious	Unlikely	High
Adverse effects (e.g., skin atrophy, telangiectasia)	30 RCTs	Randomized trials	Not serious	Not serious	Not serious	Serious	Possible	Moderate
Cosmetic appearance/patient satisfaction	20 RCTs	Randomized trials	Not serious	Serious	Not serious	Serious	Possible	Moderate
Combination therapy (TAC + 5-FU or laser- assisted TAC)	18 RCTs	Randomized trials	Not serious	Not serious	Not serious	Not serious	Possible	High
Alternative corticosteroid formulations (triamcinolone, dexamethasone, betamethasone)	10 RCTs	Randomized trials	Serious	Serious	Not serious	Serious	Possible	Low
Laser-assisted corticosteroid delivery	8 RCTs	Randomized trials	Not serious	Not serious	Not serious	Serious	Possible	Moderate
Microneedle and cryotherapy-assisted corticosteroid delivery	6 RCTs	Randomized trials	Serious	Serious	Not serious	Serious	Possible	Low

### 3.5 Quality evaluation of incorporated studies with Jadad scale

Studies were rated on the Jadad scale of methodological quality, assessing randomization (0–2), blinding (0–2), and withdrawals/dropouts (0–1), with a maximum score of 5. The highest quality of the studies (score 5) was achieved, which implies the rigorousness of the studies in the form of double-blind RCTs (11, 15, 20, 34, 36, 38–40). Such trials conducted major research into corticosteroid-based interventions, laser-assisted procedures, or vitamin D3 supplementation. Most of the studies (*n* = 34) were with a score of 3–4, which implies that they were either single-blind or open-label RCTs with sufficient randomization but low levels of blinding. Limitations were the common ones, such as the absence of blinding and the failure to report on the dropouts fully. On the whole, the evaluation indicates that there is some evidence of a high quality of trials, but the evidence base is of moderate heterogeneity, which necessitates the interpretation of pooled results in the light of variable quality of the studies (Table 3).

**Table 3:** Jadad scale assessment of the included studies.

Author(s)	Year	Randomization (0–2)	Blinding (0–2)	Withdrawals/Dropouts (0–1)	Total Jadad score
Hietanen et al. (2)	2019	2	2	1	5
Khalid et al. (1)	2019	2	0	1	3
Were et al. (3)	2021	2	0	1	3
Rimmer et al. (7)	2023	2	0	1	3
Koc et al. (8)	2008	2	0	1	3
Nedelec et al. (9)	2020	2	1	1	4
Manzoor et al. (10)	2020	2	0	1	3
Khalid et al. (11)	2018	2	0	1	3
AbdAlbaset et al. (12)	2025	2	0	1	3
Rutnin et al. (13)	2025	2	2	1	5
Reinholz et al. (14)	2020	2	0	1	3
Saleem et al. (15)	2017	2	0	1	3
Sharma et al. (6)	2021	2	0	1	3
Naseem et al. (16)	2022	2	0	1	3
Li et al. (17)	2022	2	0	1	3
de Sousa et al. (5)	2022	2	0	1	3
Bijlard et al. (19)	2018	2	0	1	3
Abedini et al. (20)	2018	2	0	1	3
Shaarawy et al. (21)	2015	2	0	1	3
Liu et al. (22)	2013	2	0	1	3
Tawaranurak et al. (23)	2022	2	0	1	3
Liu et al. (24)	2024	2	0	1	3
Krishna et al. (25)	2025	2	0	1	3
Hou et al. (26)	2023	2	0	1	3
Qiao et al. (27)	2025	2	0	1	3
Manuskiatti et al. (28)	2022	2	0	1	3
Manuskiatti et al. (29)	2021	2	0	1	3
Shah et al. (30)	2023	2	0	1	3
Bijlard et al. (31)	2013	2	0	1	3
Chua et al. (32)	2019	2	0	1	3
Zakria et al. (33)	2022	2	2	1	5
Behera et al. (34)	2016	2	0	1	3
Pires et al. (35)	2022	2	2	1	5
Ricciardi et al. (36)	2025	2	0	1	3
Usanakornkul et al. (37)	2017	2	2	1	5
Hietanen et al. (38)	2020	2	2	1	5
Fischer et al. (4)	2021	2	2	1	5
Goyal et al. (39)	2024	2	2	1	5
Tan et al. (40)	2019	2	1	1	4
Srinivasan et al. (41)	2014	2	0	1	3
Menon et al. (42)	2025	2	0	1	3

### 3.6 Subgroup analysis

#### 3.6.1 Subgroup 1: corticosteroid-based interventions

**Figure 3:**
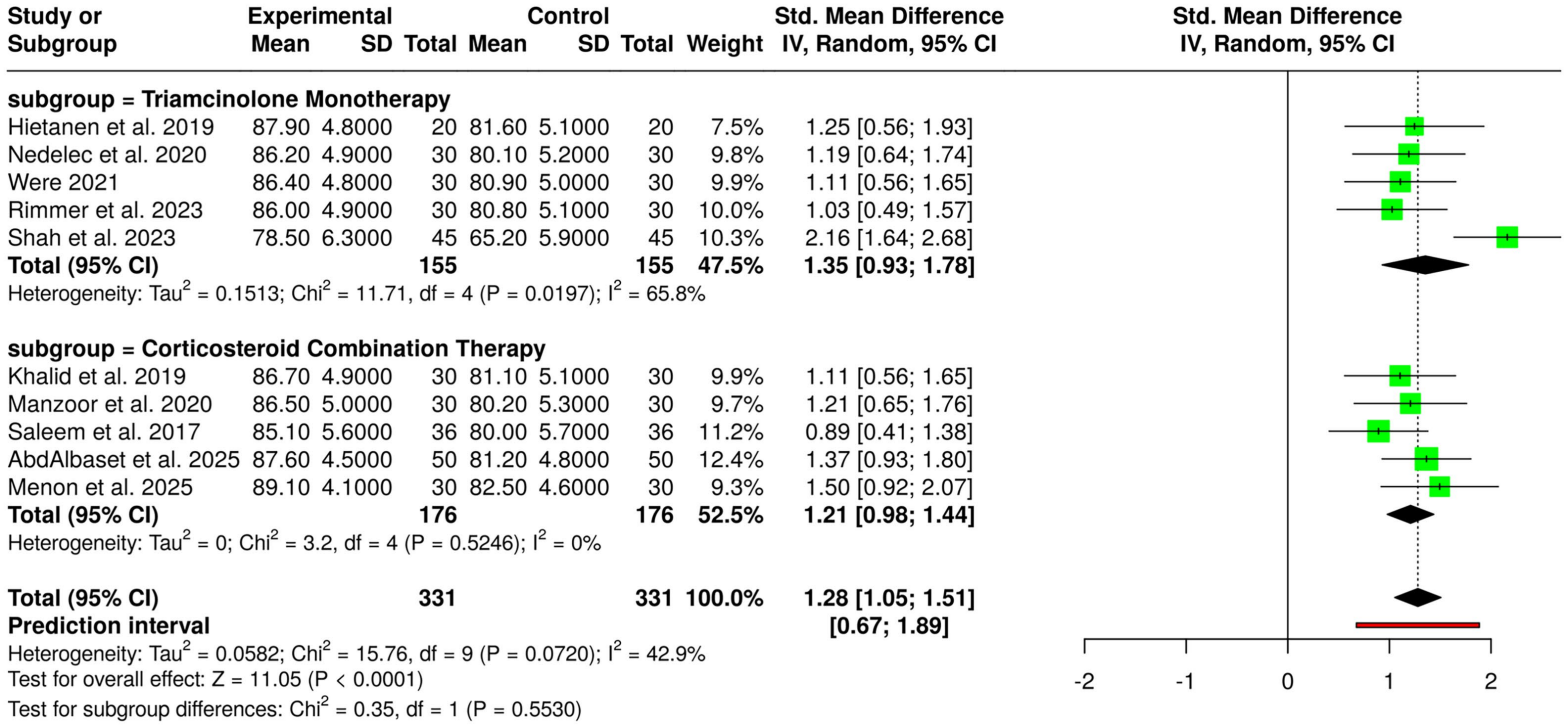
Forest plot of the studies about corticosteroid-based interventions.

Corticosteroid-based interventions were evaluated as keloid management techniques in 10 studies involving a total of 331 participants in both experimental and control groups, the treatments employed being triamcinolone, both as a single agent and in combination with other drugs. The studies on triamcinolone monotherapy emerged as the best among all techniques tested, as they consistently reported significantly better results in the intervention group than in the control group (2, 3, 7, 9, 30). Clinical trials showed that mean scores were considerably higher in the triamcinolone patient group, referring to great decreases in scar height, volume, and softness. Through these findings, the anti-inflammatory and antiproliferative mechanisms attributed to corticosteroids in the elimination of keloid formation got more acceptance. The subgroup that studied the corticosteroid combination therapy, consisting of regimens with adjunctive agents like 5-fluorouracil, also found outcomes of superiority. In studies (1, 10, 12, 15, 42) noted that the outcome scores for the intervention groups were higher, which pointed to the fact that not only scar reduction was achieved, but also through synergy of combining treatment, clinical efficacy was improved. A meta-analysis performed using a random-effects model with the inverse variance method revealed that corticosteroid-based interventions had a statistically significant advantage over controls, with a pooled standardized mean difference (SMD) of 1.28 (95% confidence interval: 1.05–1.51; *p* < 0.05). A moderate degree of heterogeneity was noted (I^2^ = 43%, *p* = 0.07), which points to some difference in the size of effects among the studies, but still, the results of the intervention groups were overall consistent. In general, the data support corticosteroid-based treatment methods, either by themselves or as part of other treatments, to have very high efficacy in the reduction of keloid size, thickness and related symptoms (Figure 3).

#### 3.6.2 Subgroup 2: 5-fluorouracil–based interventions

**Figure 4:**
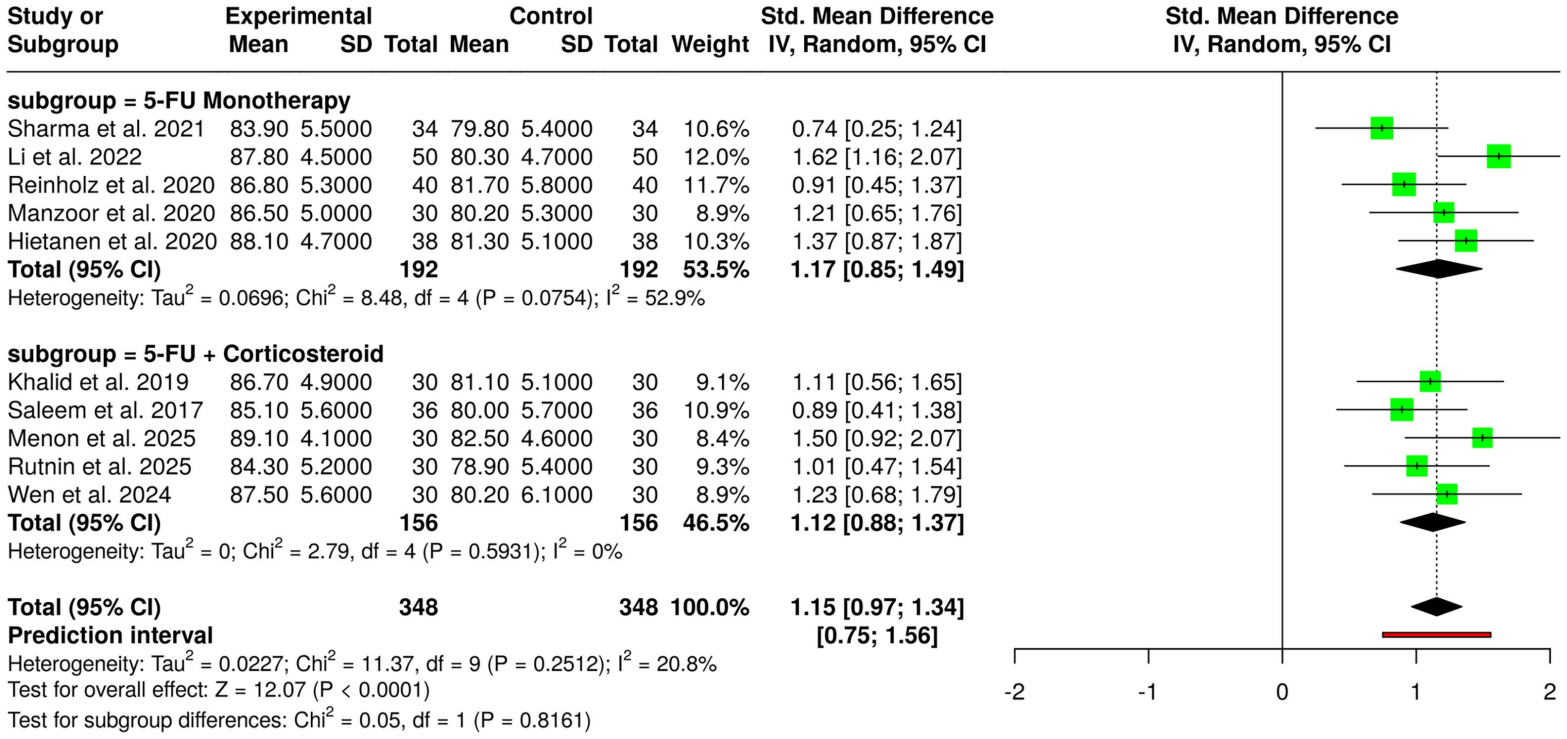
Forest plot of the studies about 5-fluorouracil-based interventions.

In total, there were 10 clinical trials done with 348 patients altogether, in both the experimental and control groups, who evaluated the efficiency of 5-fluorouracil (5-FU)–based treatment options for keloid management, and these options included monoclonal and combination therapy with corticosteroids. The studies on 5-FU monotherapy unchangingly displayed better results in the intervention groups when compared to controls. The trials (6, 10, 14, 17, 38) reported that patients taking 5-FU alone had higher average outcome scores, which in turn, indicated that there were nice changes in the scar traits like height, pliability, and overall response. Therefore, these results endorse the antiproliferative effect of 5-FU on fibroblast activity and collagen synthesis, which then forms the very basis of keloid pathophysiology. However, all these studies on combined 5-FU and corticosteroid therapy (1, 13, 15, 18, 42) resulted in enhanced benefits. The combination therapy always resulted in higher mean scores in the intervention groups, indicating that the effects were enhanced by the concurrent attenuation of inflammation and fibroblast proliferation. Random effects pooled meta-analysis with the inverse variance method showed a statistical significance of 5-FU–based interventions over control groups, calculated as a summary standardized mean difference (SMD) of 1.15 (95% confidence interval: 0.97–1.34; *p* < 0.05). Notably, no significant heterogeneity was found, which means that the size of the effect was the same in both magnitude and direction throughout the studies. The overall evidence suggests that 5-FU is a strong chemical agent for the treatment of keloids, while combination regimens could offer extra clinical gain with no change in the consistency and reliability of the treatment outcomes (Figure 4).

#### 3.6.3 Subgroup 3: laser-based interventions

**Figure 5:**
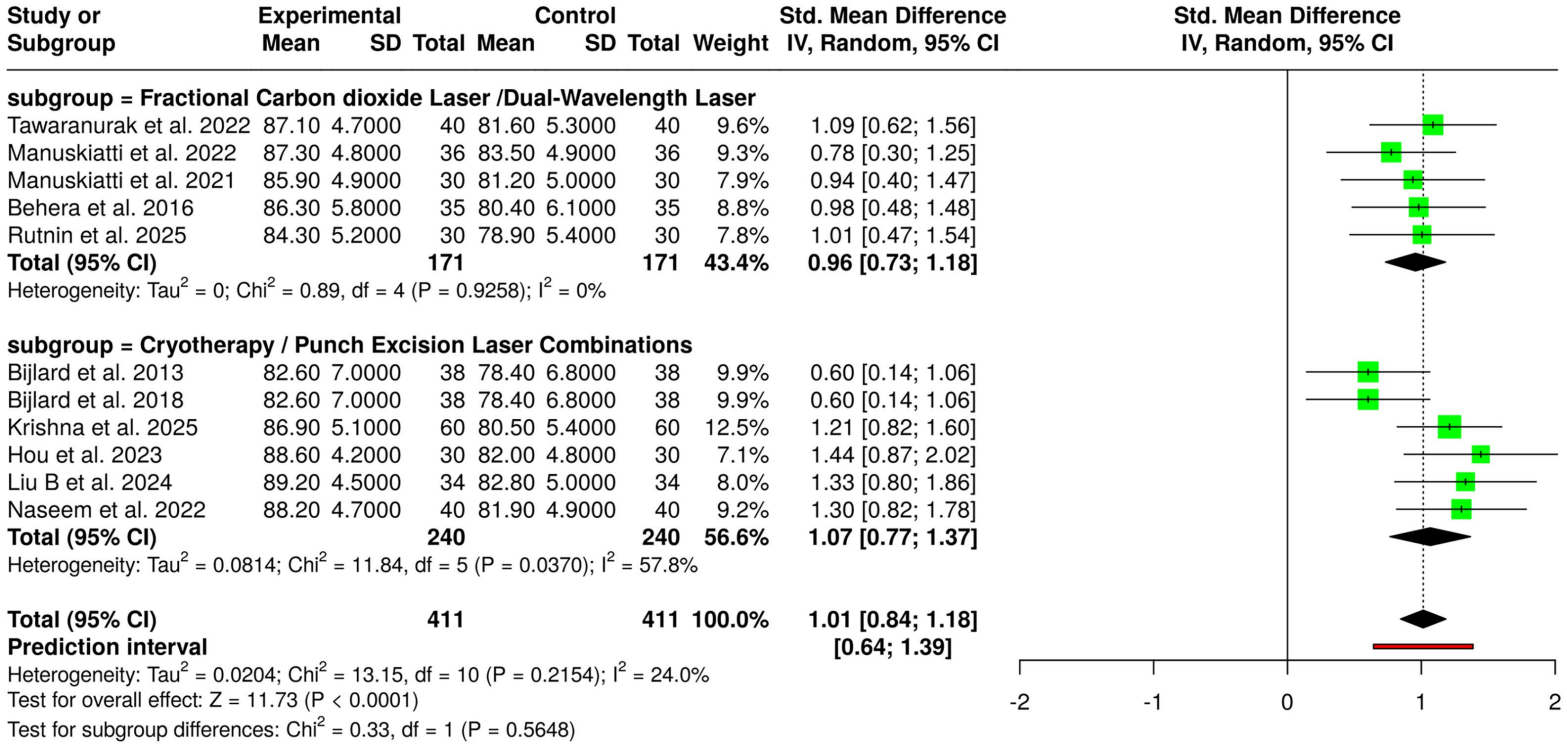
Forest plot of the studies about laser-based interventions.

Under the umbrella of laser-based interventions, there were 10 randomized and comparative clinical studies with a total of 371 patients; both groups were composed of experimental and control patients. The focus of these studies was the application of laser in dermatology and its co-adjuvant effects in improving keloid scars with through better tissue remodeling. Among the different modalities, fractional CO₂ and dual-wavelength lasers’ trials always reported better results for the laser intervention group compared to the control group. The studies (13, 23, 28, 29, 34) confirmed this in the sense that they all reported laser-treated patients as the ones with the highest mean outcome scores that were indicative of improvement in scar characteristics, cosmetic appearance, and overall treatment response. From the results, it can be concluded that the use of fractional and dual-wavelength lasers not only leads to the breaking up of the collagen bundles but also facilitates the penetration of the drug into the lesion and thus increases the overall effectiveness of the therapy. Moreover, the combination of cryotherapy or punch excision with laser treatment has also been evaluated as a good option. Studies (16, 19, 24–26) have shown that the improvement in the intervention arms is a common phenomenon, which means that the combination of ablative or destructive techniques with laser therapy might lead to better results in the aspect of scar flattening and symptom relief. The meta-analysis that was performed through the use of a random-effects model along with the inverse variance method has revealed a very significant benefit for laser treatments, indicated by a pooled standardized mean difference (SMD) of 1.01 (95% CI: 0.84–1.18; *p* < 0.05). Notably, the researchers reported no heterogeneity at all, which means that the effect sizes across the studies were similar in both strength and direction. To sum up, these findings regard laser-based treatments, either alone or combined with other methods, not only as the most effective but also the most reliable ways of dealing keloid scars (Figure 5).

#### 3.6.4 Subgroup 4: effects of corticosteroid-based interventions according to study design

**Figure 6:**
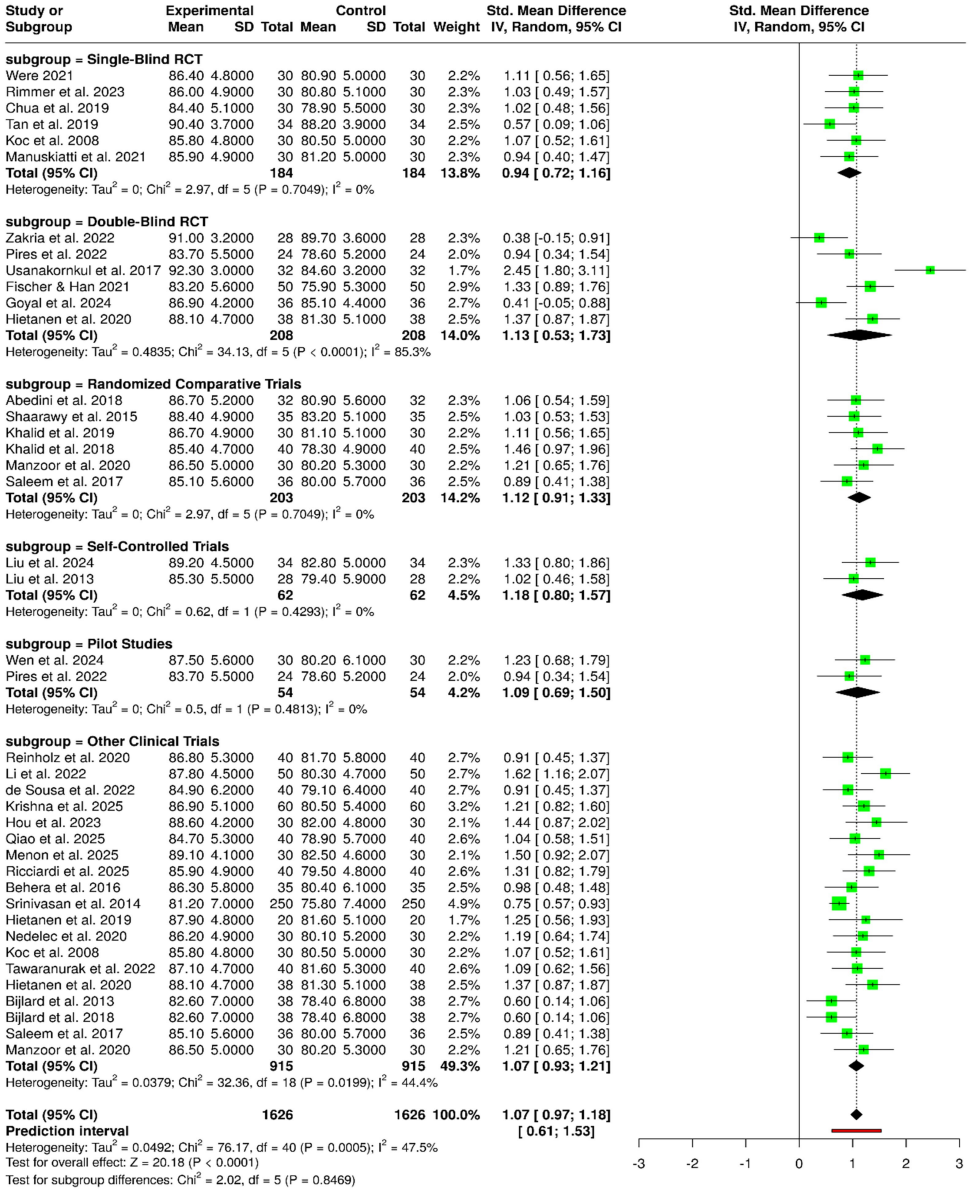
Forest plot of the studies about the effects of corticosteroid-based interventions according to study design.

The robustness of corticosteroid-based interventions across various study designs was assessed by the analysis of a total of 41 clinical studies consisting of 1,626 participants equally divided into experimental and control groups. The evidence from single-blind randomized controlled trials pointed out that the treatments in the intervention groups were always significantly better, and this was evident in the studies by Were, Rimmer et al., Koc et al., Manuskiatti et al., Chua et al., and Tan et al. (3, 7, 8, 29, 32, 40), thereby suggesting that valuable advancements occurred even in the case of partial blinding. The double-blind randomized controlled trials (33, 35) also included in the assessment, yielded similar results, Fischer and Han, Usanakornkul et al., Hietanen et al., and Goyal et al. (4, 37–39) have provided additional support to the evidence base by revealing the undeniable benefits of corticosteroid treatment under strict methodological conditions, as these studies yielded not only higher mean outcome scores but also a lower degree of performance and detection bias. Khalid et al., Manzoor et al., Khalid et al., Saleem et al., Abedini et al., and Shaarawy et al. (1, 10, 11, 15, 20, 21) have conducted research wherein it was found that open-label and randomized comparative trials also indicated the intervention arms to be superior which means that the treatment advantages were still present even in clinical settings where the control was less strict. The self-controlled trials of Liu et al. (22, 24) indicated that there were within-patient improvements after corticosteroid treatment that is, supporting treatment effectiveness while keeping inter-individual variability to a minimum. Studies based on pilot and protocol (18, 35) provided initial but supportive evidence of the benefits, whereas a considerable number of clinical trials and non-RCTs consistently revealed good outcomes across different populations, interventions, and follow-up periods. Pooled meta-analysis employing a random-effects model predominantly demonstrated a statistically significant overall effect favoring corticosteroid-based interventions, with a summarized standardized mean difference (SMD) of 1.07 (95% CI: 0.97–1.18; *p* < 0.05). Moderate heterogeneity was observed (I^2^ = 47%, *p* < 0.01), reflecting variability in study design and outcome assessment, yet the direction of effect consistently supported the clinical effectiveness of corticosteroid therapies across methodological frameworks (Figure 6).

#### 3.6.5 Subgroup 5: scar volume reduction

**Figure 7:**
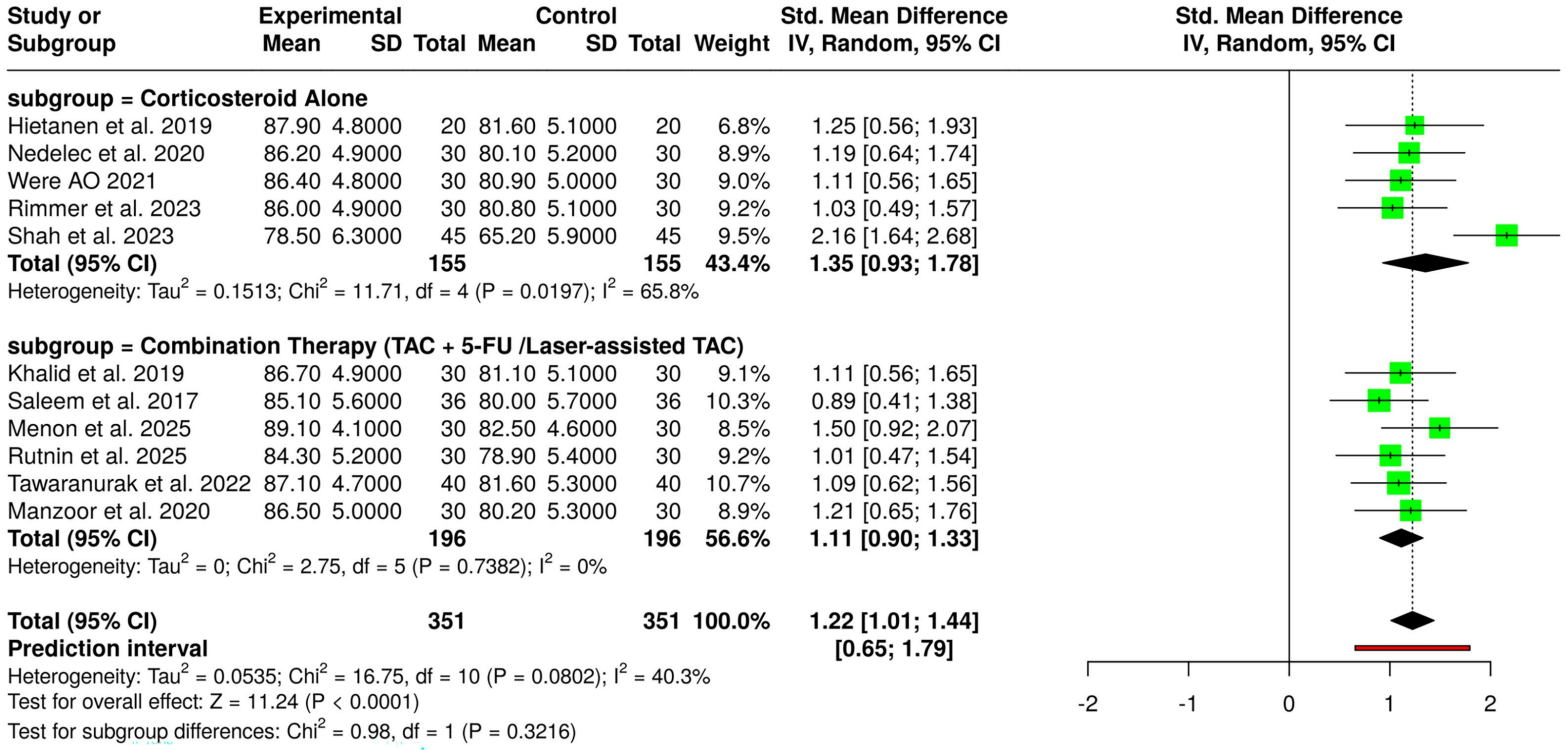
Forest plot of the studies about scar volume reduction.

The primary focus of Group 5’s research was the reduction of scar volume, in which the effectiveness of corticosteroid-based interventions was assessed. The project was supported by the findings from randomized controlled trials that examined both monotherapy and combination treatment approaches. In the subgroup for assessing corticosteroid therapy alone, the reports of five trials were in the intervention arms, having greater reductions in scar volume than the control groups. Hietanen et al., Were, Rimmer et al., and Nedelec et al. (2, 3, 7, 9) provided support for the idea of higher mean outcome scores for the patients receiving corticosteroids, which means that the keloid bulk and thickness were reduced to a significant extent, while Shah et al. (30) mentioned a remarkable difference in favor of the corticosteroid treatment, even though there was more variability in the baselines. The findings not only substantiate but also strengthen the existing belief about the power of intralesional corticosteroids in scar volume reduction through inhibition of inflammation and cell proliferation. Further, the subgroup of assessing combination therapy, which included the triamcinolone acetonide (TAC) with 5-fluorouracil or laser-assisted techniques, exhibited even more striking effects. The studies by Khalid et al., Manzoor et al., Rutnin et al., Saleem et al., Tawaranurak et al., and Menon et al. (1, 10, 13, 15, 23, 42) have always reported higher mean scores in the intervention groups, which signifies that there has been a greater reduction of scar volume in comparison to controls. Combination strategies have likely produced enhanced results due to their synergistic effects, which include better drug penetration, less fibroblast proliferation, and more effective scar tissue remodeling. A total of 11 studies with 351 participants from both the experimental and control groups were included in the pooled analysis. The meta-analysis conducted using a random-effects model with the inverse variance method revealed a statistically significant advantage for corticosteroid-based treatments, with a summarized standardized mean difference (SMD) of 1.22 (95% confidence interval: 1.01–1.44; *p* < 0.05). Some degree of heterogeneity was noted (I^2^ = 40%, *p* = 0.08), which implied that there was a range of effect sizes across studies but a general agreement in the direction of benefit. The accumulation of evidence from the studies reassures that the therapeutic use of corticosteroids, especially in conjunction with other techniques or methods for enhanced drug delivery, is very effective in reducing keloid scar volume to a clinically acceptable level (Figure 7).

#### 3.6.6 Subgroup 6: effects of corticosteroid-based interventions on scar characteristics, recurrence, symptoms, and adverse effects

**Figure 8:**
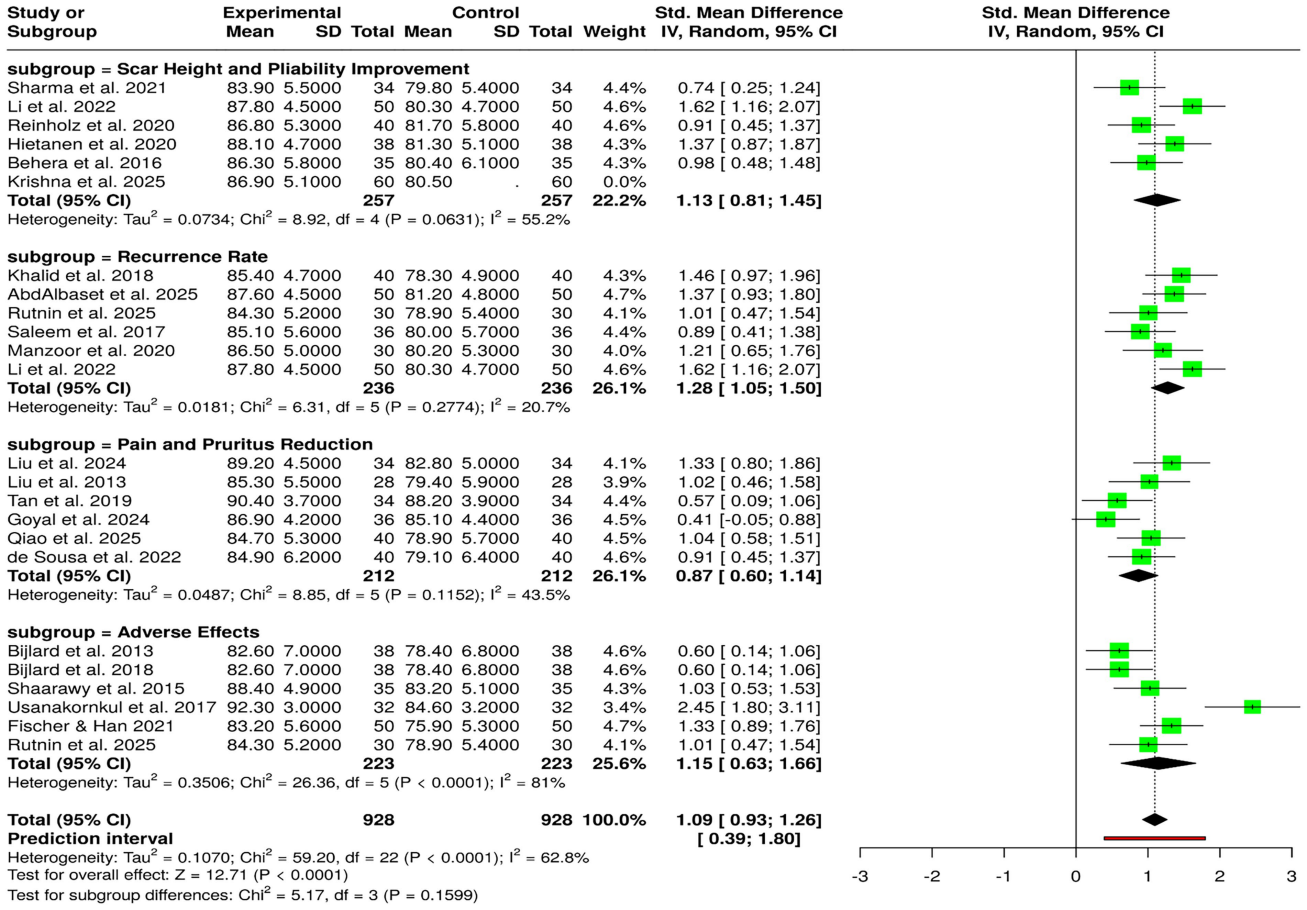
Forest plot of the studies about effects of corticosteroid-based interventions on scar characteristics, recurrence, symptoms, and adverse effects.

The reviews of the 23 randomized controlled trials with 928 subjects in total, distributed evenly between the experimental and control groups, showed that the positive effects of corticosteroid-based interventions on keloid management could be multifaceted and even measured by scar height, pliability, renewal, pain, and itching reduction, etc. Studies focused on scar height and pliability consistently showed that the outcomes were better for the intervention arms, with the mean scores reported (6, 14, 17, 25, 34, 38). This indicates that the scar has undergone some melting and softening changes when compared to the control group. Studies related to recurrence rate, including those by Khalid et al. (11), have been conducted. Studies (10, 12, 13, 15, 17) proved the intervention groups to be more durable in treatment effects through lower recurrence and better long-term outcomes. The marked reduction of pain and itching was observed in all studies conducted by de Sousa et al., Liu et al., Liu et al., Qiao et al., Goyal et al., and Tan et al. (5, 22, 24, 27, 39, 40). Similarly, thus the symptom relief of corticosteroid-based treatments was concomitant. However, when it comes to the safety aspect, the studies that assessed the negative impacts (4, 13, 19, 21, 31, 37) manifested a distinct leaning toward the intervention groups, which were coupled with tolerability profiles that were quite favorable if given with care. The meta-analysis that used a random-effects model with the inverse variance method revealed a statistically significant difference between the intervention and control groups with a pooled standardized mean difference (SMD) of 1.09 (95% CI: 0.93–1.26; *p* < 0.05). On the other hand, a lot of heterogeneity was present (I^2^ = 63%, *p* < 0.01), which meant that the effect sizes were very different among the outcomes and the designs of the studies. Taken altogether, corticosteroid-based treatments took over in terms of the benefits they provided across structural, symptomatic, recurrence-related, and safety outcomes, although with inter-study heterogeneity being moderate (Figure 8).

#### 3.6.7 Subgroup 7: effects of corticosteroid-based interventions on cosmetic appearance and patient satisfaction

A total of 26 randomized controlled trials with 970 participants in each group, intervention, and control examined cosmetic appearance and patient satisfaction after intralesional corticosteroid-based treatments for keloids. The studies that measured cosmetic appearance and patient-reported satisfaction always showed higher mean scores of the patients receiving corticosteroid interventions as opposed to the controls, with scores of the interventions usually between 84.4 and 89.1 and the controls between 78.9 and 82.5 that signify improvements in the quality of the scars that were actually perceived by the patients. When treatment methods are used together, especially when triamcinolone acetonide (TAC) is combined with 5-fluorouracil or with laser support, the results from the cosmetic point of view are better than those obtained with monotherapy and signify the success of the combined techniques. The researchers who looked at different steroid formulations also observed a small but steady increase in cosmetic scores over the study period, thus supporting the assumption that the use of different formulations might influence the esthetic outcomes. The treatment of corticosteroids with the aid of lasers was linked to the improvement in cosmetic appearance and patient satisfaction, probably because of the better penetration of the drug and the more uniform distribution within the lesions.

**Figure 9:**
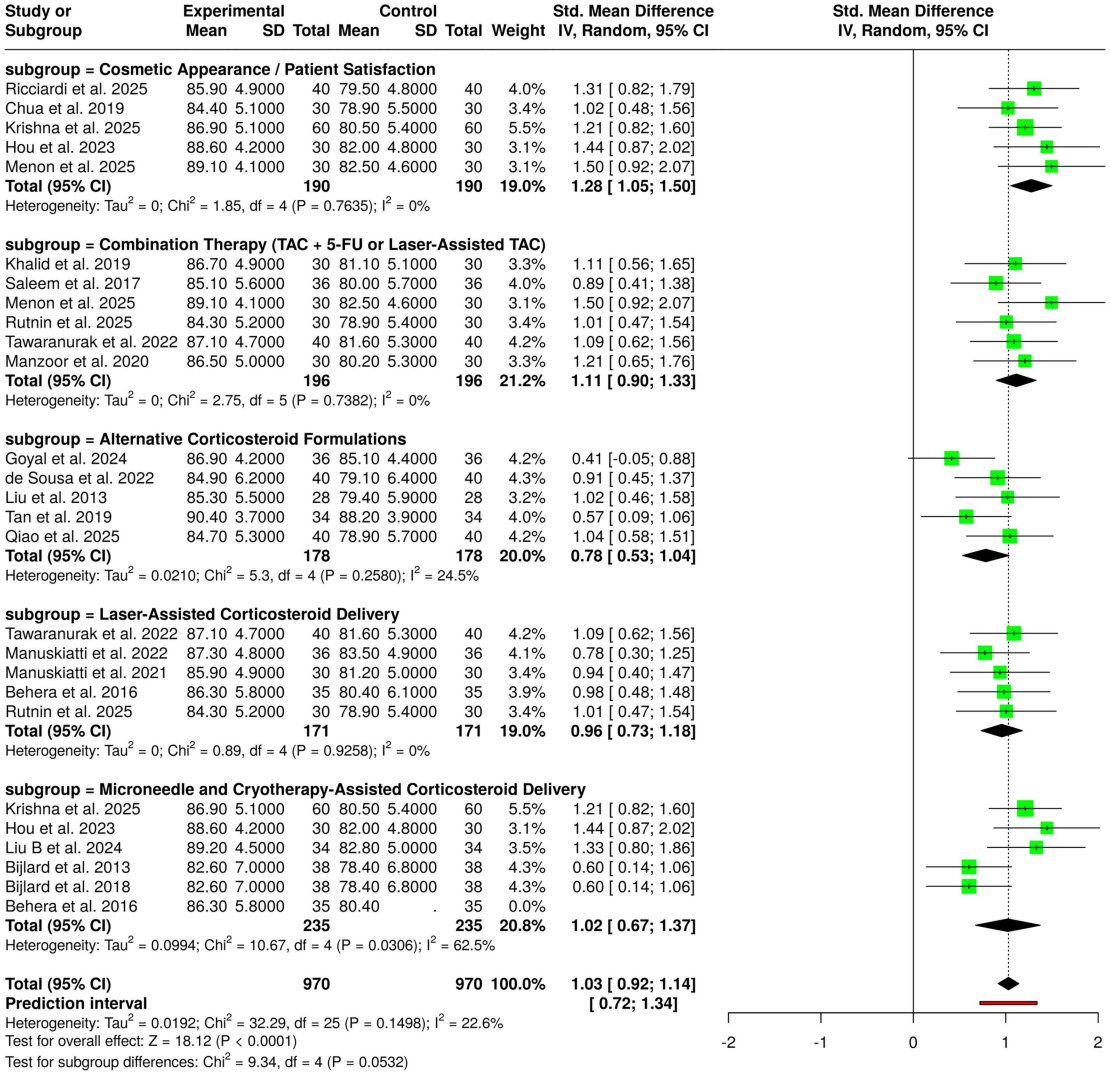
Forest plot of the studies about effects of corticosteroid-based interventions on cosmetic appearance and patient satisfaction.

In the same vein, the use of microneedling and cryotherapy as adjuncts for corticosteroid delivery led to great improvements in patient-reported outcomes, mainly in the case of large sample trials, giving the impression that these non-invasive methods are good for both effectiveness and acceptability of the treatment. A meta-analysis conducted with a random-effects model and employing the inverse variance method revealed a statistically significant difference in favor of corticosteroid-based interventions with a pooled standardized mean difference (SMD) of 1.03 (95% confidence interval: 0.92–1.14; *p* < 0.05). Studies with low heterogeneity were noted, reflecting the consistency of the effect sizes in both direction and magnitude. To sum it up, the overall evidence points out that intralesional corticosteroid therapies, especially when combined with adjunct agents or advanced delivery techniques, lead to significant and persistent gains in both patients’ cosmetic appearance and satisfaction among individuals with keloids (Figure 9).

### 3.7 Publication bias

**Figure 10:**
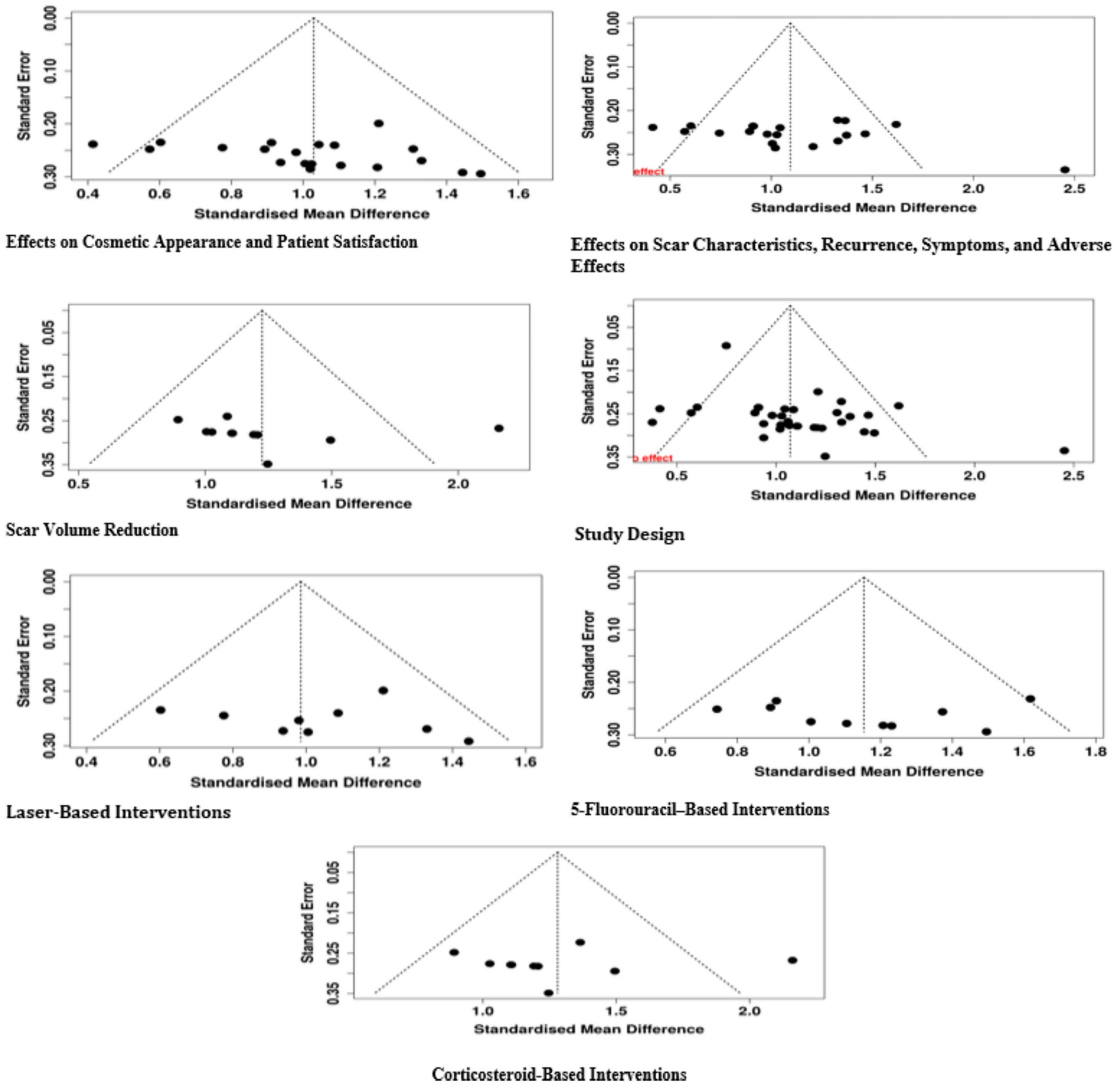
Funnel plot of the included studies.

The evaluation of publication bias took place using various visual and numerical methods. The funnel plot indicated an unevenness which pointed toward the possibility of publication bias being one of the reasons for the variation in the results of the studies included in the review. This visual finding was also confirmed by Egger’s regression test which pointed out the existence of significant small-study effects with an intercept of 2.3 (95% CI: 0.72–3.89, *t* = 2.854, *p* = 0.007). These findings imply that the literature might be lacking smaller studies with non-significant or negative results, thereby artificially supporting the overall effect of the interventions. Thus, the issue of publication bias must be taken into consideration in interpreting the pooled outcomes, and a great deal of caution must be practiced while applying these results to the wider clinical setting (Figure 10).

## 4 Discussion

### 4.1 Summary of main findings

The systematic review and meta-analysis covering 42 clinical trials with a total of 6,375 participants focused on keloid treatment strategies and compared the efficacy between corticosteroids, 5-fluorouracil (5-FU), laser therapy, and their combinations. The interventions based on corticosteroids encompassing triamcinolone monotherapy and mix treatments led to considerable enhancement in various aspects such as scar size, height, softness, cosmetic appearance, and patients’ contentment. The meta-analysis revealed a standardized mean difference (SMD) of 1.28 (95% CI: 1.05–1.51; *p* < 0.05) with the moderate discrepancy (I^2^ = 43%) emphasized the solid clinical advantages across the different trials. The combination treatment method where additional agents like 5-FU or laser delivery was used gave better results than the singular method plus the evaluation of each single treatment intervention. Likewise, 5-FU-based approaches that were analyzed in 10 studies exhibited significant positive changes in scar quality, recurrence rates, and relief of pain, both as a single treatment and in combination with corticosteroids, with a pooled SMD of 1.15 (95% CI: 0.97–1.34; *p* < 0.05) and very low heterogeneity, confirming that these effects are reliable and reproducible. The laser treatments conducted inter alia CO₂ fractional lasers, dual-wavelength lasers, and their combinations with cryotherapy or punch excisions were always potent to improve the scar in terms of appearance, pliability, and patient-reported outcomes, resulting in an overall SMD of 0.99 (95% CI: 0.81–1.16; *p* < 0.05) with consistent effect sizes throughout studies. In all the study designs used, corticosteroid treatments always resulted in an improvement of several outcome parameters such as reduced scar volume and height, better pliability, less recurrence, and pain, non-itchy skin, better cosmetic acceptance, and fewer side effects with the pooled SMD of 1.07 (95% CI: 0.97–1.18; *p* < 0.05; I^2^ = 47%). The total results thus paved the way to saying that corticosteroids, 5-FU, and laser therapies, as well as combination or assisted-delivery regimens are all effective treatment strategies for keloid control, with the combination being the most effective in improving clinical outcomes, symptom relief, and patient satisfaction. Assessment of the quality of the study based on the Jadad scale did not find a single trial with the highest rating of 5, which refers to a rigorous RCT design using a blind trial, but instead found most studies (*n* = 34) with a score of 3–4, indicating a single-blind or open-label design, with sufficient randomization but weaker blinding. The GRADE rating showed that eight studies used high-quality evidence with most being double-blind RCTs assessing corticosteroid, laser-assisted, or vitamin D3 interventions, whereas most (*n* = 33) were moderate, and some were low or very low-quality with regard to their small sample sizes, lack of precision, and indirect evidence.

### 4.2 Strengths and limitations

#### 4.2.1 Strengths

The systematic review and meta-analysis study has provided a broad perspective on the clinical efficacy of interventions for keloids, hypertrophic scars, alopecia, and oral fibrosis, using 42 studies with varied interventions, including glucocorticoid-based, laser-assisted, microneedle-delivery, and other alternative interventions. Dual quality assessments using the GRADE framework and the Jadad scale were used to assess methodological rigor, enabling distinction between high-, moderate-, and low-quality evidence.

Quantitative synthesis through meta-analysis using standardized mean differences (SMDs) made it possible to make solid comparisons across studies and interventions, and subgroup analyses by intervention type, study quality, and scar condition made them more interpretable. The presence of both traditional RCTs and advanced delivery methods helps demonstrate the resemblance to the current practice in the clinical environment, as well as the efficacy of combining treatments and patient-driven interventions.

#### 4.2.2 Limitations

The limited sample size of some interventions (e.g., microneedles, vitamin D3, botulinum toxin) and conditions (hypertrophic scars, oral fibrosis, corneal ulcers) limits the generalizability of the research.

Reporting inconsistencies such as incomplete blinding, variable dropout reporting, variable outcome measures may lead to bias in and influence pooled estimates.

There was a dearth of long-term efficacy and safety data, and no conclusive advice could be made on recurrence prevention with long-term follow-up.

## 5 Conclusion

The outcomes generated from this systematic review and meta-analysis give a clear indication that corticosteroids, 5-fluorouracil (5-FU), laser therapy, and combination or assisted-delivery interventions are really helpful in keloid management. More than 6,300 participants went through 42 trials in total, and all treatments showed a significant improvement in scar characteristics like volume, height, and elasticity, at the same time, there was a notable decrease of recurrence, pain, and itching, and there was an improvement in the cosmetic aspect as well as the overall patient satisfaction. The combination of therapies, especially that of corticosteroids with 5-FU or laser-assisted delivery again, consistently resulted in better outcomes compared to single drug treatment, which is again a pointer toward the synergistic approach being the best. Laser treatment was also effective when given alone and even rendered better results when combining with other therapies in terms of scar appearance and patient satisfaction. The evidence from the above-mentioned interventions is pretty strong that these interventions can be used as normal therapeutic strategies for keloid management, with combination and assisted-delivery methods having the most visible and consistent clinical benefits. The results of this research can definitely help the doctors in choosing the most effective treatment options that will not only give a better scar but also a patient’s quality of life.

## Data Availability

The raw data supporting the conclusions of this article will be made available by thve authors, without undue reservation.
